# The effects of neighborhood perceptions on response to a technology-assisted parenting intervention for adolescent substance use: protocol of a diversity supplement to parent SMART (Substance Misuse in Adolescents in Residential Treatment)

**DOI:** 10.1186/s13722-024-00509-y

**Published:** 2024-10-18

**Authors:** Zabin Patel-Syed, Sarah A. Helseth, Robert Rosales, Tim Janssen, Kelli Scott, Sara J. Becker

**Affiliations:** 1 Department of Psychiatry and Behavioral Sciences, Center for Dissemination and Implementation Science, Northwestern University Feinberg School of Medicine, Chicago, USA; 2 Department of Behavioral and Social Sciences, Brown University School of Public Health, Providence, USA; 3 Department of Psychiatry and Behavioral Sciences, Brown University School of Public Health, Providence, USA; 4 Department of Medical Social Sciences, Center for Dissemination and Implementation Science, Northwestern University Feinberg School of Medicine, Chicago, USA

**Keywords:** Substance use, Neighborhoods, Technology-assisted, Intervention, Parenting, Adolescent

## Abstract

**Background:**

It is well established that an adolescent’s neighborhood is associated with their likelihood of developing a substance use disorder. The availability of drugs, lack of access to resources, and exposure to violence are all associated with greater substance use among young people, leading to more pronounced health inequities. Technology assisted interventions (TAIs) have been touted to enhance the reach of substance use treatment and improve outcomes for high-need families living in underserved neighborhoods. A key question is whether neighborhood characteristics impact the effectiveness of TAIs, given these interventions are embedded within an adolescent’s natural environment. This National Institute on Drug Abuse-funded Diversity Supplement will examine the role of perceived neighborhood characteristics on response to Parent SMART, a TAI for parents of adolescents in residential substance use treatment (R37DA052918; PI: Becker). Aim 1 will use both adolescent and parent self-report of multiple neighborhood dimensions (e.g., physical environment, social disorder, satisfaction with community resources) to identify indicators predictive of treatment response. Aim 2 will then explore the indirect relationship between neighborhood context and response to Parent SMART, via engagement.

**Methods:**

Participants include adolescent and parent dyads enrolled in an effectiveness trial evaluating Parent SMART, a TAI for parents of adolescents in residential substance youth treatment. Participants will complete self-report measures of neighborhood physical environment, social disorder, and satisfaction with community resources at baseline to predict parenting and youth substance outcomes at 6-, 12-, and 24-weeks post discharge.

**Discussion:**

To date, few studies have explicitly tested how neighborhood affects response to TAIs for adolescent substance use. Assessing adolescent and parent perceptions of neighborhood characteristics holds potential to pinpoint key contextual factors that affect TAI response and to promote consideration of multi-level health equity determinants in substance use research. Understanding neighborhood influences can advance public health by helping tailor TAIs to address the unique needs of adolescents living in underserved communities.

**Trial registration:**

This study extends the measurement and analysis plan of a pragmatic effectiveness trial. The pragmatic effectiveness trial is registered at ClinicalTrials.gov NCT05169385; https://clinicaltrials.gov/ct2/show/NCT05169385

**Supplementary Information:**

The online version contains supplementary material available at 10.1186/s13722-024-00509-y.

## Background

Neighborhoods confer both risk and protective factors for adolescent substance use. Drug availability, limited access to resources, and exposure to violence predict greater substance use among young people and worsen extant health inequities [1, 2]. Nurturing neighborhoods can play a critical role in positive youth development and decrease the likelihood of drug use by providing adolescents access to social supports, recreational activities, and safe public spaces that reduce stress [3]. Neighborhood, a well-established social determinant of health, broadly encompasses a community’s physical and socioeconomic environment [3, 4], [5]. Although neighborhoods provide critical context for adolescent substance use behaviors, neighborhood-level variables (e.g., physical environment, social disorder, access to resources) are rarely measured in clinical trials of behavioral health interventions [6]. Exploring the role of neighborhoods in intervention research can provide a more complete understanding of the multi-level factors that influence treatment processes and outcomes [7].

**Fig. 1:**
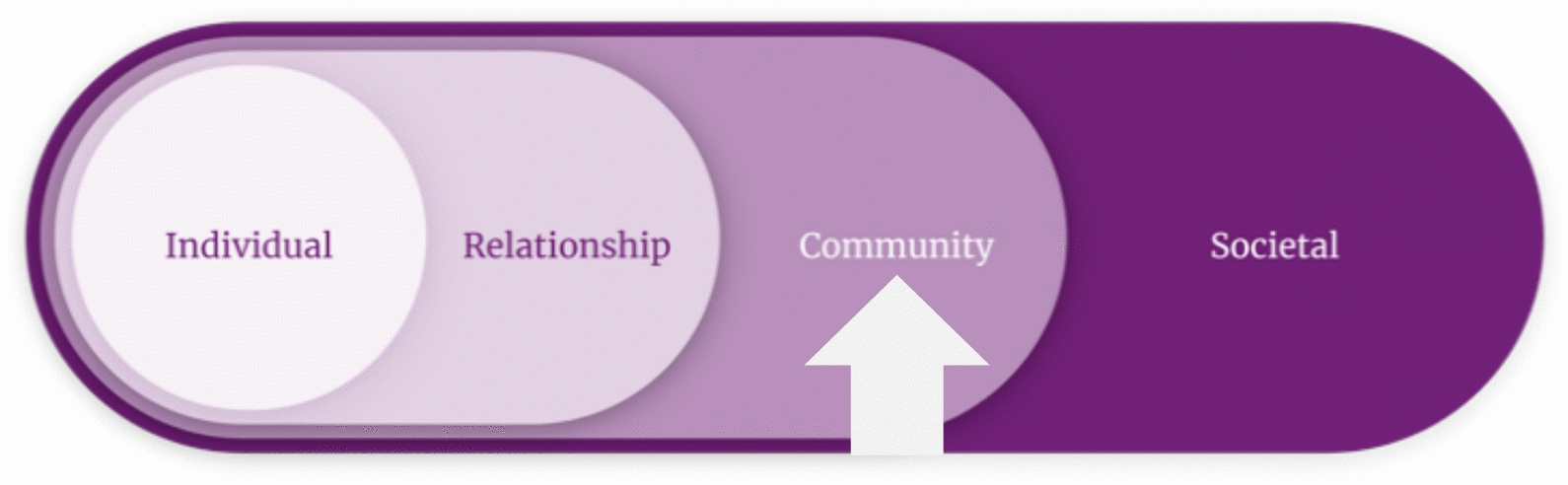
The socioecological model emphasizes multiple levels of influence on adolescent substance use behavior. The parent grant examines the “Individual” and “Relationship” levels on intervention outcomes. This protocol will extend the aims of the parent grant via measurement and analysis of “Community” level variables (e.g., neighborhood) on response to the Parent SMART intervention

The social ecological model illustrates how neighborhoods exert influence on individual health behaviors [8]. This public health framework posits that adolescent substance use is best understood situated within multiple overlapping contexts, including individual- (e.g., sociodemographic), relationship- (e.g., family), community- (e.g., neighborhood), and societal- (e.g., policy) level influences (Fig. 1). For example, living in a neighborhood with high crime can increase an adolescent’s stress levels and risk for mental health problems, elevating the risk of substance use as a coping strategy [9, 10]. Conversely, living in a neighborhood with strong social supports may help an adolescent feel more connected to peers and lead to more frequent contact with positive role models, which can buffer against substance use [1]. Although associations between neighborhood and substance use are well known [11, 12], the role of neighborhood variables in treatment engagement and response remains largely unexplored.

Technology-assisted interventions (TAI) may be especially useful in improving access to effective behavioral healthcare for families living in underserved neighborhoods. A major advantage of TAIs for substance use is that they are embedded within an individual’s natural environment, thereby circumventing several structural barriers to accessing care including poor public transportation, limited availability of trained providers, and the stigma associated with help seeking [13, 14]. Additionally, TAIs can be delivered and scaled at low cost, while TAIs are more costly to develop compared to traditional clinic services, delivery costs per patient decrease significantly over time, increasing the potential for cost effectiveness [15]. Given TAIs provide adolescents and families on demand access to evidence-based substance use care, there is a growing push to expand TAI implementation in underserved communities [16, 17].

Neighborhood characteristics, which are majorly understudied, may impact response to a TAI. The compounding stress of living in an underserved neighborhood can increase adolescent risk for substance use *and* lower effectiveness of TAIs [2, 3]. Living in an underserved community may reduce a family’s ability to access, engage with, and consequently benefit from intervention components. Although TAIs offer timely support, stressors associated with living in underserved neighborhoods may challenge a family’s capacity to participate in TAI activities (e.g., watch video content, respond to text alerts, attend scheduled live coaching sessions) and gain therapeutic benefit (e.g., parenting skill acquisition, symptom relief, reduction in substance use). Existing health inequities, coupled with consistently high adolescent substance use rates [18] compel us to scrutinize intervention response in at-risk populations like underserved communities [19]. Incorporating measures of neighborhood variables in clinical research more fully acknowledges the environments in which adolescents and families live and the contexts in which TAIs for substance use are delivered.

Explicitly considering neighborhood variables in the evaluation of TAIs can help ensure such interventions are effective in reducing health disparities in high-need areas and equitably advancing public health. However, neighborhood variables often go unmeasured in intervention research for several reasons: (a) integrating clinical data with neighborhood data from government sources (e.g., U.S. Census) requires specialized training; (b) there is lack of consensus among researchers on which aspects of neighborhood to assess; and (c) it is unknown whose perspective is most crucial to consider when multiple family members are engaged in treatment [6, 8, 20]. Examining neighborhood perceptions using multiple dimensions and respondents can improve our understanding of TAI response in underserved communities and promote more nuanced consideration of how to measure multi-level health equity determinants in substance use research.

This protocol describes a Diversity Supplement funded by the National Institute on Drug Abuse embedded within a pragmatic effectiveness trial, which will explore the role of neighborhood context on response to a parenting TAI. We briefly describe the aims of the pragmatic effectiveness trial, the aims of the Diversity Supplement, and the Diversity Supplement’s methods. Additional sections of the Diversity Supplement, including the candidate’s Career Development Plan and Mentoring Plan, can be found in Supplemental Materials.

### Pragmatic effectiveness trial aims

Improving outcomes after discharge from adolescent residential substance use treatment is critical to ensure continuity of care and prevent relapse [21, 22]. Involving parents improves adolescent retention and outcomes in substance use treatment, but parents often face structural barriers to care access including high costs and provider shortages [23, 24]. Thus, targeting parents through low-burden and easily accessible TAIs has the potential to enhance the quality of adolescent substance use treatment [22, 25]. The randomized trial in which the current Diversity Supplement is embedded tests the effectiveness of a parenting TAI called Parent SMART (Substance Misuse among Adolescents in Residential Treatment, [26] among 220 adolescent-parent dyads in residential substance use treatment. Parent SMART consists of three main intervention components: (a) a scalable online program, Parenting Wisely, with demonstrated efficacy in improving parenting skills and decreasing youth behavior problems [27], (b) four telehealth coaching sessions to tailor Parenting Wisely content to each family’s needs, and (c) a smartphone app-based networking forum where parents can connect with other parents that have teens in substance use treatment, ask experts questions, and receive daily support text messages [28]. Parent SMART is initiated during the adolescent’s residential stay and delivery continues following discharge to support transition into community care and prevent relapse. In a prior pilot study of 61 parent-adolescent dyads (R34DA039298), Parent SMART demonstrated high feasibility, acceptability, and initial effectiveness in improving parental communication, enhancing parental monitoring, decreasing adolescent drinking, and reducing school problems [25, 29].

Building upon the successful pilot trial, the pre-registered Specific Aims of the pragmatic effectiveness trial (R37DA052918) are to evaluate the effectiveness of Parent SMART relative to residential treatment as usual (TAU) on: (a) proximal parenting outcomes, defined as improvement in parental monitoring and communication (Primary Aim 1); (b) distal adolescent substance use outcomes, defined as reductions in substance use and substance-use related problems (Primary Aim 2); and (c) distal adolescent problem behaviors, including school problems, externalizing behaviors, sexual risk behaviors, and criminal involvement (Secondary Aim). An exploratory study aim is to examine the extent to which change in parenting processes mediates change in adolescent substance outcomes.

### Diversity supplement aims

**Fig. 2:**
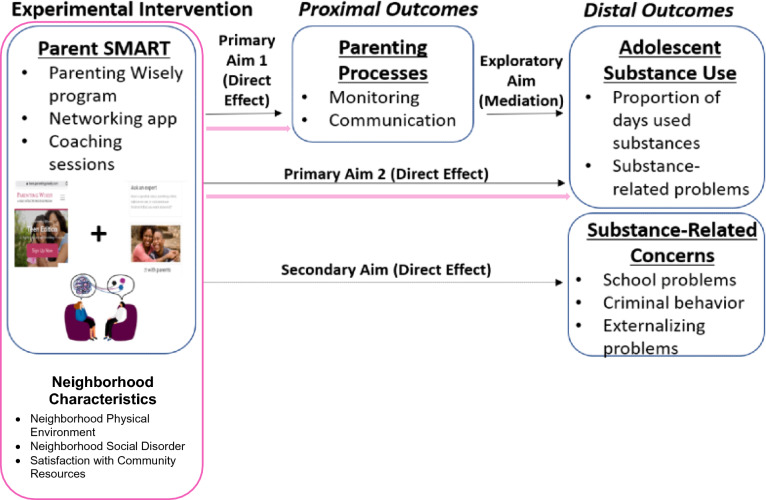
Research aims of the current Diversity Supplement are highlighted. Research aims of the parent grant R37DA052915 (PI: Becker) are shown in blue

The current protocol will assess the role of parent and adolescent neighborhood perceptions on response to Parent SMART, a TAI for parents of adolescent in residential substance use treatment [30]. We will extend the pragmatic effectiveness trial’s assessment and analysis plan by examining whether neighborhood perceptions uniquely predict parenting and youth substance use outcomes beyond individual- and family-level sociodemographic factors (Fig. 2). Exploring neighborhood effects can help us determine whether specific modifications to Parent SMART are needed to enhance intervention potency for families living in under-resourced neighborhoods.

This protocol advances previous studies of neighborhood effects on substance use interventions, which have generally used epidemiological samples, relied on U.S. Census data, and conducted retrospective analyses [3]. Prior approaches have also been limited by measurement error [31] and failed to consider that individuals living in the same zip code could have fundamentally different perspectives of neighborhood resources [32]. We will address these limitations by conducting a multi-respondent, multi-dimensional evaluation of adolescent and parent neighborhood perceptions.

This protocol has two Specific Aims. Specific Aim 1 will examine the extent to which neighborhood context, assessed via multiple respondents and dimensions, affects response to the Parent SMART intervention. Specific Aim 1 tests the hypothesis that neighborhood disadvantage will predict poorer response to Parent SMART as measured by (1a) parental monitoring and communication and (1b) adolescent days of substance use and substance-related problems at 6-, 12- and 24-weeks. The effect of neighborhood context will persist even after controlling for individual- (e.g., race/ethnicity, gender, and age) and family-level variables (e.g., income and household size).

Specific Aim 2 will investigate the indirect relation between neighborhood context and response to Parent SMART, via engagement. Specific Aim 2 tests the hypothesis that engagement with Parent SMART at 6 weeks will indirectly affect the relationships between neighborhood context and intervention outcomes, as measured by parental monitoring and communication and adolescent days of substance use and substance-related problems at 12 and 24 weeks.

## Methods

### Participants

Inclusion criteria for parents in the ongoing pragmatic effectiveness trial include: (a) parent or legal guardian of an adolescent aged 12–18 years in residential treatment due to substance use; (b) will be primary guardian living with adolescent after their discharge from residential treatment; (c) English or Spanish fluency; and (d) willing to complete a structured assessment prior to adolescent’s discharge from residential treatment. Adolescents whose parents meet inclusion criteria are eligible if they assent to research and complete a structured assessment prior to discharge from residential treatment. All parents and adolescents who are eligible for the pragmatic effectiveness trial are eligible for the current protocol and will complete the additional neighborhood measures. The pragmatic effectiveness trial launched in 2023 and will recruit 220 parent-adolescent dyads. At the time protocol received ethics approval, 80 dyads were enrolled. We expect to enroll all of the 140 remaining dyads for the current study.

Based on our team’s prior work in residential settings [25, 29], parents are expected to be mostly biological mothers (80%). Adolescent participants are expected to self-identify as approximately 45% male, 45% female, and 10% nonbinary. We anticipate the sample to self-identify as the following racial groups: 65% White, 12% Black, 15% Multiracial, and 2% Asian (we expect that about 6% will “prefer not to answer” about their racial identity), we also expect a quarter (25%) of the sample to identify their ethnicity as Hispanic.

### Procedures

Enrollment, randomization, and follow-up procedures for the current protocol are embedded within the pragmatic effectiveness trial and described in a published protocol [30]. Briefly, parents are referred to the study by the partner facility’s intake coordinator, who informs parents about their eligibility and provides them with a consent to contact form if interested in participating. The consent to contact form gives an overview of the study and gathers the parent’s contact information for later outreach by research staff, who conduct a brief study screen with the parent. Eligible parents consent to study participation via an electronic consent form, provide parental consent for their child, and complete a 45–60-min baseline assessment. Research staff collaborate with residential staff to arrange a time for the adolescent to learn about the study via Zoom or phone call. Interested adolescents complete an eligibility screen, provide informed consent via electronic forms, and complete a 90–120-min baseline assessment. Parents and adolescents must independently provide consent for a dyad to enroll in the study.

After completing the baseline assessment, parent-adolescent dyads are randomly assigned to receive residential TAU or TAU and Parent SMART. Urn randomization [33] balances adolescent biological sex at birth (male versus female) and parent’s preferred language (English versus Spanish). Parents are informed of the assignment immediately after the baseline assessment. Research staff conduct follow-up assessments with parents and adolescents at 6, 12, and 24 weeks after discharge from residential treatment. Parents and adolescents are each compensated up to $200 for completing study assessments.

### Study conditions

#### Residential TAU

TAU consists of standard residential treatment provided to adolescents at our partner facility, Rosecrance Health Network. Services will be received by families in both study conditions. Recommended length of stay is 42 days on average, but treatment is tailored to every adolescent’s needs. Adolescents at Rosecrance Health Network participate in approximately 15 h of educational programming and 25 h of treatment weekly, primary in group therapy based on dialectic behavioral therapy [34]. Additionally, adolescents are assigned a primary counselor who conducts check-ins twice a week. Medication management is provided by a psychiatrist or nurse practitioner as required. Before discharge, parents receive a tailored relapse prevention plan and either a referral to an outpatient therapy provider or a return or previous outpatient provider. Families are also provided with a referral to a psychiatrist or self-help groups as needed. After study randomization, parents are given an education pamphlet about substance use, parenting skills, and local community resources, consistent with usual resource provision at the partner site.

#### Parent SMART

Parents randomized to TAU and Parent SMART receive a TAI consisting of three core components, which were developed based on formative feedback from the pilot trial [26]. All components are available in English and Spanish and described in detail elsewhere [30].

*Online Parent Skills Program.* Parents receive a 24-week subscription to Parenting Wisely, a self-administered online parenting program shown to reduce youth problem behaviors across several trials [35–37]. Parenting wisely consists of nine modules corresponding to common family problems (e.g., finding drugs in the home). Each module includes a video depicting a family struggling with a specific problem, parents choose from three potential vignette solutions, only one of which is an effective approach. Parents receive feedback on their chosen solution, which highlights using communication and monitoring skills (I Statements, Reflective Listening, Contracting, Asking Key Question) and why their selected solution was effective or ineffective. Parenting Wisely takes 3–5 h to complete and is accompanied by a workbook that outlines the modules, includes sample behavior plans, and provides parents with practice exercises.

*Networking Forum.* Parents receive access to a private, moderated networking forum with two main channels, Ask an Expert and Connect with Parents, accessible via a smartphone app or web browser. The Ask an Expert channel allows parents to ask questions to a team of clinical psychologists and the Connect with Parents channel allows parents to pose questions and comments to other parents of adolescents in residential treatment. The smartphone app sends daily push notifications with a “Tip of the Day!” providing encouragement, reminders to use the forum, and links to Parenting Wisely videos. The forum also includes parenting resources, information about the study team, and is available in English and Spanish.

*Coaching Sessions.* Parents are offered up to four telehealth coaching sessions to tailor the Parenting Wisely program to a family’s needs and to promote engagement in the networking forum. Each session follows an outline consisting of an explanation of a specific parenting skill (I Statements, Reflective Listening, Contracting, Asking Key Question); review of a Parenting Wisely module; role play of the session’s parenting skill; and opportunity to ask questions, share concerns, and receive feedback. The first coaching session is 60–75 min and introduces parents to Parent SMART before covering the outline; the remaining three sessions are 45–60 min. Parents receive homework in between sessions, which includes completing Parenting Wisely modules that map onto their unique concerns and to using the networking forum for additional support. Coaching sessions are delivered by trained BA-level or MA-level research staff under the supervision of a licensed clinical psychologist.

Sessions are audio recorded and a random subset of at least 25% of sessions are reviewed for adherence and competence by an independent evaluator on the study team; 25% of those selected for coding are double coded. Adherence is rated via a study specific adherence checklist that ranges from 0 (no elements covered) to 17 (all elements covered), with a target of 80%. Competence is rated via the 6-item “General Therapeutic Skills” subscale of the Cognitive Therapy Rating Scale [38]. Items are scored on a 6-point Likert scale, with a higher score indicating greater competence,a mean score of ≥4 is the competence target.

### Measures: pragmatic effectiveness trial

Table 1 presents an overview of study measures by timepoint. The following measures are collected as part of the pragmatic effectiveness trial’s assessment battery and will be used as outcome measures for the Diversity Supplement. These measures will be collected at baseline, 6, 12, and 24-week follow-up assessments.

**Table 1.** Overview of study measures by timepoint

	Timepoint			
	Baseline	6-week	12-week	24-week
Pragmatic effectiveness Trial Measures				
Parental Monitoring Questionnaire (PMQ; [39])	X	X	X	X
Parent-Adolescent Communication Scale (PACS; [40])	X	X	X	X
Family Assessment Task (FAsTask)	X	X	X	X
Global Appraisal of Individual Needs (GAIN; [41])	X	X	X	X
Additional diversity supplement measures				
Physical Environment (PNDS; [4])	X			
Social Disorder (PNDS; [4])	X			
Satisfaction with Community Resources	X			

Note: Measures for the current Diversity Supplement protocol will be collected from adolescent and parent participants at the baseline assessment to predict change in parenting processes and treatment engagement at the post discharge assessments

#### Parental monitoring questionnaire

The Parental Monitoring Questionnaire (PMQ; [39] is a 24-item measure that assesses parent oversight of an adolescent’s activities (e.g., awareness of whereabouts, knowledge of friends, adherence to household rules). The measure has three subscales: child disclosure, parent solicitation, and parental control. Adolescent report of parent behavior and parent self-report are collected.

#### Parent–adolescent communication scale

The Parent–Adolescent Communication Scale (PACS; [40] measures the quality and effectiveness of communication between parents and adolescents (e.g., ability to listen, understand perspectives, resolve conflicts). Adolescent report of parent behavior and parent self-report are collected.

#### Family assessment task

The Family Assessment Task (FAsTask; [42] is an audio recorded family problem solving activity that is used to provide an in vivo assessment of parenting skills [43]. The assessment includes three 5 min tasks: (1) Parent leads a discussion about setting limits and adolescent reacts, (2) Parent leads a discussion on household rules about substance use and adolescent reacts, (3) Adolescent leads a discussion about a time they were unsupervised with their peers and then parents react. Each task is rated by a trained research study member masked to study condition on limit setting, parent substance use beliefs, parent substance use communication, adolescent disclosure, and parental monitoring based on a structured codebook adapted for the parent study [44, 45]. Ratings between 1 and 3 indicate poor parenting skills, ratings greater than 3 and less than 6 indicate average parenting skills, and ratings 6 and above indicate strong parenting skills.

#### Substance use

Adolescent substance use and substance use related problems are assessed via the Global Appraisal of Individual Needs, a structured clinical interview (GAIN; [41] that evaluates adolescent functioning across eight life domains: background (e.g., individual-level and family sociodemographic variables), substance use (past 90 days), mental health, physical health, risk behaviors, legal, vocational, and environmental.

### Additional measures: diversity supplement

The current protocol integrates neighborhood measures into the parent study’s baseline assessment. Both parent and adolescent self-reports of neighborhood perceptions will be collected at baseline only. Neighborhood measure selection was guided by formative work exploring the role of community-level variables on treatment response in clinical settings [6].

#### Physical environment

Perceptions of a neighborhood’s physical environment will be assessed with the Physical Disorder (4 items; “There is a lot of graffiti in my neighborhood”) and Physical Order (2 items; “My neighborhood is clean”) subscales of the Ross-Mirowsky Perceived Neighborhood Disorder Scale (PNDS; [4], a widely used measure in social science research. The physical environment component of the PNDS specifically focuses on neighborhood aspects that may be perceived as deteriorated, unkempt, or in despair (e.g., litter, graffiti, abandoned buildings). Items are scored on a 4-point Likert scale such that a higher score indicates greater disorder in the environment.

#### Social disorder

Perceptions of social behaviors and interactions within the neighborhood will be measured via the Social Disorder (5 items; “There is too much drug use in my neighborhood”) and Social Order (4 items; “My neighborhood is safe”) subscales of the Ross-Mirowsky Perceived Neighborhood Disorder Scale (PNDS; [4]). Social disorder generally encompasses items related to community experiences that may be perceived as threatening, disruptive, or unsafe (e.g., crime, gang presence, and violence. Items are scored on a 4-point Likert scale so that a higher score indicates greater neighborhood social disorder.

#### Satisfaction with community resources

Participant perceptions about the availability of resources and services (e.g., educational resources, healthcare, social services, recreational activities, internet) within their community will be measured via a five-item questionnaire developed for this study. Items are scored on a 4-point Likert scale and summed, with a higher score indicating greater satisfaction with community resources.

### Data analysis plan

**Fig. 3:**
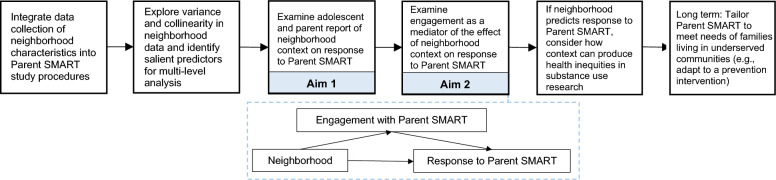
Specific Aims and analytic plan of the current protocol, a National Institute of Drug Abuse funded Diversity Supplement to 37DA052915 (PI: Becker)

The data analysis plan is illustrated in Fig. 3. Descriptive statistics (mean, standard deviation, range) will be used to examine neighborhood variable scale scores and the primary outcome variables, including parenting processes and adolescent substance use. *t*-tests for continuous variables and *chi*-square tests for categorical variables will test for differences in neighborhood perceptions between intervention groups (i.e., TAU versus TAU and Parent SMART) at baseline.

Specific Aims 1 and 2 compare trajectories of change using data from baseline, 6-, 12-, and 24-weeks follow-up assessments on parenting processes and adolescent substance use. Latent growth curve modeling will be used to examine neighborhood effects on response to the Parent SMART intervention. Various growth curve models (linear, quadratic, logarithmic, hyperbolic, and piecewise) will be examined to determine the model with the best unconditional fit to the data [46].

For Aim 1 (To examine the extent to which neighborhood context affects response to Parent SMART), differences between the two treatment groups (TAU versus TAU and Parent SMART) will be tested by conditioning the latent growth curve on a neighborhood variable. To conduct a multi-respondent, multi-dimensional analysis and identify which neighborhood dimensions and whose report is most predictive of outcome, a comprehensive model building approach will be applied to data from all dimensional scales and both reporters to estimate model parameters. Neighborhood variables will be entered as predictors in a set of nested models and changes in model fit will be examined. If neighborhood variables are highly correlated, a latent factor of neighborhood perceptions will be generated and used as a predictor for analyses. The same model building approach will be used per reporter.

For Aim 2 (To explore the indirect relationship between neighborhood context and response to Parent SMART, via engagement), engagement indicators will be examined by regressing growth curves on these engagement indicators (b-path) and regressing the engagement indicators on the neighborhood context variables (a-path). Significance of a statistical mediation pathway will be examined using the bootstrapped distribution of products (a-path * b-path) test [47].

## Discussion

The assumption that TAIs equitably improve treatment outcomes for families living in underserved communities is rarely tested empirically. This protocol paper describes a National Institute of Drug Abuse funded Diversity Supplement focused on understanding the role of neighborhood characteristics in adolescents’ and parents’ ability to engage in and benefit from Parent SMART, a parenting TAI for adolescents in residential substance use treatment. Expanding the pragmatic effectiveness trial’s assessment and analytic plan to examine neighborhood effects on TAI engagement and outcomes is the first step to understanding intervention response in underserved communities and promotes the consideration of multi-level health equity determinations in substance use research.

This study has several strengths. First, the pragmatic effectiveness trial in which this protocol is embedded is testing a low-intensity, highly scalable TAI that requires limited training to deliver. As the TAI is scaled out, understanding neighborhood context may inform potential intervention adaptations (e.g. tailoring Parent SMART to be delivered as a preventative intervention). Second, empirically examining neighborhood effect on intervention response recognizes the interplay between individual, environmental, and social factors on substance use behavior and recovery. Lastly, as the Parent SMART intervention is brought to scale, an ecological lens can help identify disparities in intervention outcomes across different neighborhoods, while highlighting specific neighborhood factors for which additional resources may be needed to target inequities.

Study limitations should also be considered. A key limitation is that this study relies on self-report. Although self-report neighborhood measures are more feasible for intervention developers to integrate into clinical trials than geocoding residential addresses, self-report is susceptible to several biases. To mitigate these biases, we also plan to conduct an exploratory analysis integrating participant clinical data with neighborhood-level U.S. Census data (e.g., zipcode or block-group data collected from the American Community Survey) to conduct a multimethod assessment of neighborhood influence on Parent SMART response. However, due to the small sample size it is possible that too few U.S. defined geographies may be represented, which would limit variability in the neighborhood-level predictors. Regardless, a comprehensive approach to assessing neighborhoods is necessary for drawing appropriate inferences and informing decision-making in resource allocation for substance use prevention efforts.

Second, after accounting for individual- and family-level factors, the magnitude of a neighborhood effect on intervention response may be modest. Additionally, despite efforts to control for confounding factors, there may be several unmeasured neighborhood-level variables that influence both TAI engagement and response. Given little is known about the importance of measuring neighborhood perceptions in clinical research, this study is a step forward in applying the socioecological model in the development and testing of TAIs.

In line with the goals of a National Institute of Drug Abuse Diversity Supplement, the research activities described above are complemented by a career development plan that will support the candidate (protocol lead author) in achieving the research aims and attaining the proficiencies necessary to pursue a career as an independent investigator (see Supplemental Materials). The career development goals for this Diversity Supplement include building expertise in three domains: (a) conducting pragmatic clinical effectiveness trials of TAIs with adolescent substance use populations; (b) evaluating the influence of TAIs on equitable access to and engagement in substance use care; and (c) using multi-level models for longitudinal data to analyze response to TAIs. The candidate (first author) will achieve these goals via individualized mentorship from a skilled mentor team with expertise in adolescent substance use treatment (SJB), TAIs (SH), health equity (RR), and advanced statistical methods (TJ). Mentorship will be augmented via workshop and seminar training in substance use interventions, healthcare technologies, implementation science, and grant writing. Achieving these career development goals will support the PI in launching an independent research program and further consideration of neighborhood context in adolescent substance use research.

## Supplementary Information

Additional file 1

## Data Availability

No datasets were generated or analysed during the current study.

## Abbreviations

TAI: Technology-assisted intervention
Parent SMART: Substance misuse in adolescents in residential treatment
TAU: Treatment as usual

## References

[1] Cambron C, Kosterman R, Rhew IC, Catalano RF, Guttmannova K, Hawkins JD. Neighborhood structural factors and proximal risk for youth substance use. Prev Sci. 2020;21(4):508–18. 10.1007/s11121-019-01072-8. 31853720 10.1007/s11121-019-01072-8 PMC7166144

[2] Swan JE, Aldridge A, Joseph V, Tucker JA, Witkiewitz K. Individual and community social determinants of health and recovery from alcohol use disorder three years following treatment. J Psychoactive Drugs. 2021;53(5):394–403. 10.1080/02791072.2021.1986243. 34727839 10.1080/02791072.2021.1986243 PMC8692391

[3] Diez Roux AV, Mair C. Neighborhoods and health. Ann N Y Acad Sci. 2010;1186(1):125–45. 10.1111/j.1749-6632.2009.05333.x. 20201871 10.1111/j.1749-6632.2009.05333.x

[4] Ross CE, Mirowsky J. Neighborhood disadvantage, disorder, and health. J Health Soc Behav. 2001;42(3):258–76. 10.2307/3090214. 11668773

[5] Sampson RJ, Raudenbush SW, Earls F. Neighborhoods and violent crime: a multilevel study of collective efficacy. Science [Internet]. 1997;277(5328):918–24. 10.1126/science.277.5328.918. 10.1126/science.277.5328.918 9252316

[6] Patel-Syed Z, Moise IK, Bulotsky-Shearer R, Price M, Becker SJ, Jensen-Doss A. Conceptualizing neighborhood context in youth psychotherapy research. J Clin Child Adolesc Psychol. 2024;53:1–7. 10.1080/15374416.2024.2303705. 38407998 10.1080/15374416.2024.2303705

[7] Braveman PA, Cubbin C, Egerter S, Chideya S, Marchi KS, Metzler M, et al. Socioeconomic status in health research: one size does not fit all: One size does not fit all. JAMA [Internet]. 2005;294(22):2879–88. 10.1001/jama.294.22.2879. 10.1001/jama.294.22.2879 16352796

[8] Kumpfer KL, Turner CW. The social ecology model of adolescent substance abuse: Implications for prevention. Int J Addctn. 1990;25:435–63. 10.3109/10826089009105124. 10.3109/10826089009105124 2093088

[9] Leventhal T, Dupéré V, Brooks-Gunn J. Neighborhood influences on adolescent development. In: Handbook of adolescent psychology: Contextual influences on adolescent development, vol. 2. 3rd ed. Hoboken: Wiley; 2009. p. 411–43.

[10] Stockdale SE, Wells KB, Tang L, Belin TR, Zhang L, Sherbourne CD. The importance of social context: Neighborhood stressors, stress-buffering mechanisms, and alcohol, drug, and mental health disorders. Soc Sci Med (1982). 2007;65(9):1867–81. 10.1016/j.socscimed.2007.05.045. 10.1016/j.socscimed.2007.05.045 PMC2151971 17614176

[11] Trucco EM. A review of psychosocial factors linked to adolescent substance use. Pharmacol Biochem Behav. 2020;196: 172969. 10.1016/j.pbb.2020.172969. 32565241 10.1016/j.pbb.2020.172969 PMC7415605

[12] Zimmerman GM, Farrell C. Parents, peers, perceived risk of harm, and the neighborhood: Contextualizing key influences on adolescent substance use. J Youth Adolesc. 2017;46(1):228–47. 10.1007/s10964-016-0475-5. 27016218 10.1007/s10964-016-0475-5

[13] Marsch LA, Borodovsky JT. Technology-based interventions for preventing and treating substance use among youth. Child Adolesc Psychiatric Clin N Am. 2016;25(4):755–68. 10.1016/j.chc.2016.06.005. 10.1016/j.chc.2016.06.005 PMC5405456 27613350

[14] Schulte MHJ, Boumparis N, Huizink AC, Riper H. Technological interventions for the treatment of substance use disorders. Comprehensive Clinical Psychology. 2022;2022:264–82. 10.1016/B978-0-12-818697-8.00010-8.

[15] Tebb KP, Erenrich RK, Jasik CB, Berna MS, Lester JC, Ozer EM. Use of theory in computer-based interventions to reduce alcohol use among adolescents and young adults: A systematic review. BMC Public Health. 2016;16(1):517. 10.1186/s12889-016-3183-x. 27317330 10.1186/s12889-016-3183-x PMC4912758

[16] Chang BL, Bakken S, Brown SS, Houston TK, Kreps GL, Kukafka R, Safran C, Stavri PZ. Bridging the digital divide: Reaching vulnerable populations. J Am Med Informatics Assoc JAMIA. 2004;11(6):448. 10.1197/jamia.M1535. 15299002 10.1197/jamia.M1535 PMC524624

[17] Sugarman DE, Campbell ANC, Iles BR, Greenfield SF. Technology-based interventions for substance use and comorbid disorders: An examination of the emerging literature. Harvard Rev Psychiatry. 2017;25(3):123–34. 10.1097/HRP.0000000000000148. 10.1097/HRP.0000000000000148 PMC5421396 28475504

[18] *2019 National Survey of Drug Use and Health (NSDUH) Releases*. (2024). Retrieved February 28, 2023, from https://www.samhsa.gov/data/release/2019-national-survey-drug-use-and-health-nsduh-releases

[19] McCuistian C, Burlew K, Espinosa A, Ruglass LM, Sorrell T. Advancing health equity through substance use research. J Psychoactive Drugs. 2021;53(5):379–83. 10.1080/02791072.2021.1994673. 34706637 10.1080/02791072.2021.1994673 PMC8692385

[20] Burlew K, McCuistian C, Szapocznik J. Racial/ethnic equity in substance use treatment research: The way forward. Addctn Sci Clin Pract. 2021;16(1):50. 10.1186/s13722-021-00256-4. 10.1186/s13722-021-00256-4 PMC8340520 34353373

[21] King CA, Beetham T, Smith N, Englander H, Button D, Brown PCM, Hadland SE, Bagley SM, Wright OR, Korthuis PT, Cook R. Adolescent residential addiction treatment in the US: Uneven access, waitlists and high costs. Health Affairs. 2024;43(1):64–71. 10.1377/hlthaff.2023.00777. 38190597 10.1377/hlthaff.2023.00777 PMC11082498

[22] Passetti LL, Godley MD, Kaminer Y. Continuing care for adolescents in treatment for substance use disorders. Child Adolesc Psychiatric Clin N Am. 2016;25(4):669–84. 10.1016/j.chc.2016.06.003. 10.1016/j.chc.2016.06.003 PMC5018300 27613345

[23] Acevedo A, Harvey N, Kamanu M, Tendulkar S, Fleary S. Barriers, facilitators, and disparities in retention for adolescents in treatment for substance use disorders: A qualitative study with treatment providers. Subst Abuse Treatment Prev Policy. 2020;15(1):42. 10.1186/s13011-020-00284-4. 10.1186/s13011-020-00284-4 PMC7302144 32552836

[24] Becker SJ, Curry JF. Outpatient interventions for adolescent substance abuse: A quality of evidence review. J Consult Clin Psychol. 2008;76(4):531–43. 10.1037/0022-006X.76.4.531. 18665683 10.1037/0022-006X.76.4.531

[25] Becker SJ, Helseth SA, Janssen T, Kelly LM, Escobar KI, Souza T, Wright T, Spirito A. Parent SMART (Substance Misuse in Adolescents in Residential Treatment): Pilot randomized trial of a technology-assisted parenting intervention. J Subst Abuse Treatment. 2021;127:108457. 10.1016/j.jsat.2021.108457. 10.1016/j.jsat.2021.108457 PMC8442663 34134877

[26] Becker SJ, Hernandez L, Spirito A, Conrad S. Technology-assisted intervention for parents of adolescents in residential substance use treatment: Protocol of an open trial and pilot randomized trial. Addctn Sci Clin Pract. 2017;12:1. 10.1186/s13722-016-0067-4. 10.1186/s13722-016-0067-4 PMC5210307 28049542

[27] Pushak RE, Gordon DA. Parenting Wisely: Using innovative media for parent education. In: Evidence-based parenting education: A global perspective. London: Routledge; 2016. p. 161–75.

[28] Helseth SA, Scott K, Escobar KI, Jimenez F, Becker SJ. What parents of adolescents in residential substance use treatment want from continuing care: A content analysis of online forum posts. Subst Abuse. 2021;42(4):1049–58. 10.1080/08897077.2021.1915916. 10.1080/08897077.2021.1915916 PMC8591665 33945453

[29] Becker SJ, Helseth SA, Janssen T, Kelly LM, Escobar K, Spirito A. Parent smart: Effects of a technology-assisted intervention for parents of adolescents in residential substance use treatment on parental monitoring and communication. Evid-Based Pract Child Adolesc Mental Health. 2021;6(4):459–72. 10.1080/23794925.2021.1961644. 10.1080/23794925.2021.1961644 PMC8791644 35087933

[30] Becker SJ, Helseth SA, Kelly LM, Janssen T, Wolff JC, Spirito A, Wright T. Parent SMART (Substance Misuse in Adolescents in Residential Treatment): Protocol of a randomized effectiveness trial of a technology-assisted parenting intervention. JMIR Res Protoc. 2022;11(2): e35934. 10.2196/35934. 35225821 10.2196/35934 PMC8922142

[31] Bilheimer LT, Klein RJ. Data and measurement issues in the analysis of health disparities. Health Serv Res. 2010;45(5 Pt 2):1489–507. 10.1111/j.1475-6773.2010.01143.x. 21054368 10.1111/j.1475-6773.2010.01143.x PMC2965888

[32] Echeverria SE, Diez-Roux AV, Link BG. Reliability of self-reported neighborhood characteristics. J Urban Health Bull N Y Acad Med. 2004;81(4):682–701. 10.1093/jurban/jth151. 10.1093/jurban/jth151 PMC3455923 15466849

[33] Stout RL, Wirtz PW, Carbonari JP, Del Boca FK. Ensuring balanced distribution of prognostic factors in treatment outcome research. J Stud Alcohol. 1994;Suppl 12:70–5. 10.15288/jsas.1994.s12.70. 10.15288/jsas.1994.s12.70 7723001

[34] McCredie MN, Quinn CA, Covington M. Dialectical behavior therapy in adolescent residential treatment: Outcomes and effectiveness. Resid Treatment Children Youth. 2017;34(2):84–106. 10.1080/0886571X.2016.1271291.

[35] Cefai J, Smith D, Pushak RE. Parenting wisely: Parent training via CD-ROM with an Australian sample. Child Fam Behav Therapy. 2010;32(1):17–33. 10.1080/07317100903539709.

[36] Cotter KL, Bacallao M, Smokowski PR, Robertson CIB. Parenting interventions implementation science: How delivery format impacts the parenting wisely program. Res Soc Work Pract. 2013;23(6):639–50. 10.1177/1049731513490811.

[37] Kacir CD, Gordon DA. Parenting adolescents wisely: The effectiveness of an interactive videodisk parent training program in Appalachia. Child Family Behav Therapy. 1999;21(4):1–22. 10.1300/J019v21n04_01.

[38] Young J, Beck AT (1980) *Cognitive Therapy Rating Scale Manual*. 1–24. 10.1037/t00834-000

[39] Stattin H, Kerr M. Parental monitoring: A reinterpretation. Child Dev. 2000;71(4):1072–85. 10.1111/1467-8624.00210. 11016567 10.1111/1467-8624.00210

[40] Barnes HL, Olson DH. Parent-adolescent communication and the circumplex model. Child Dev. 1985;56(2):438. 10.2307/1129732.

[41] Dennis ML, Titus JC, White MK, Unsicker JI, Hodgkins D. Global appraisal of individual needs: Administration guide for the GAIN and related measures. Bloomington, IL: Chestnut Health Systems; 2003.

[42] Micalizzi L, Meisel SN, Thomas SA, Parnes JE, Graves H, Becker SJ, Spirito A. Psychometric properties of the family assessment task parental monitoring scenario among adolescents receiving substance use treatment. J Subst Use Addctn Treatment. 2024;158: 209232. 10.1016/j.josat.2023.209232. 10.1016/j.josat.2023.209232 PMC10947900 38061631

[43] Barker P. Intervening in adolescent problem behavior: A family-centered approach. J Can Acad Child Adolesc Psychiatry. 2006;15(2):94–5. 10.1111/j.1475-3588.2005.00125_3.x 32806797

[44] Dishion TJ, Kavanagh K. Intervening in adolescent problem behavior: A family-centered approach. New York: Guilford Press; 2003.

[45] Hernandez L, Rodriguez AM, Spirito A. Brief family based intervention for substance abusing adolescents. Child Adolesc Psychiatric Clin N Am. 2015;24(3):585–99. 10.1016/j.chc.2015.02.010. 10.1016/j.chc.2015.02.010 PMC4475574 26092741

[46] Liu S, Rovine MJ, Molenaar PCM. Selecting a linear mixed model for longitudinal data: Repeated measures analysis of variance, covariance pattern model, and growth curve approaches. Psychol Methods. 2012;17(1):15–30. 10.1037/a0026971. 22251268 10.1037/a0026971

[47] Tofighi D, MacKinnon DP. RMediation: An R package for mediation analysis confidence intervals. Behav Res Methods. 2011;43(3):692–700. 10.3758/s13428-011-0076-x. 21487904 10.3758/s13428-011-0076-x PMC3233842

