# Supplementary material for: The effects of neighborhood perceptions on response to a technology-assisted parenting intervention for adolescent substance use: protocol of a diversity supplement to parent SMART (Substance Misuse in Adolescents in Residential Treatment)
Source: Addict Sci Clin Pract. 2024 Oct 18;19:75. doi: 10.1186/s13722-024-00509-y (PMC11488280; doi:10.1186/s13722-024-00509-y)
Supplement: Supplementary file 1 — Additional file 1 [file 13722_2024_509_MOESM1_ESM.docx]

**CAREER DEVELOPMENT PLAN**

I am deeply committed to enhancing the effectiveness of substance use interventions for youth and families in underserved communities. A primary focus of my developing program of research is to understand how contextual factors, such as community-level variables, influence treatment effectiveness and implementation. TAIs show strong potential to increase access to care and improve substance use outcomes for families living in underserved neighborhoods.^14,21^ However, this assumption is rarely tested. With this Diversity Supplement, I will build the expertise required to launch an independent research program focused on leveraging technology to ensure equitable access to treatment and improve substance use outcomes for underserved youth and families. This proposal builds directly on my expertise examining treatment effectiveness in community settings by evaluating how neighborhood characteristics affect response to Parent SMART, a TAI for substance use. I will build on my interdisciplinary public health and clinical psychology background by developing proficiency in conducting pragmatic effectiveness trials of TAIs with youth who use substances, building my knowledge base on methods to evaluate the equitable delivery of TAIs, and acquiring the data analytic skills in multi-level longitudinal modeling needed to assess response to TAIs. These research and career development activities will support my ultimate career goal to become an independent researcher in an academic medical center focused on improving the delivery of substance use interventions for youth and families. Upon completion of this Diversity Supplement, I will apply for a NIDA R34 to adapt Parent SMART to better meet the needs of families from underserved neighborhoods based upon the lessons learned in this supplement. If we find that neighborhood affects response to intervention, my R34 will propose formative research with families to adapt or develop new modules of the Parent SMART intervention to better address their needs. If we do not find that neighborhood affects response to intervention, then I will work with families to adapt Parent SMART for use as a preventive or early intervention, since it is currently designed for adolescents in residential treatment. This approach could have broad applicability for adolescents earlier in the care continuum. Understanding how contextual factors may influence intervention effectiveness is a critical step prior to widespread implementation and sustainment of TAIs. In my future work, I will study strategies to increase the uptake of TAIs for substance use in underserved neighborhoods. As such, this Diversity Supplement will set the foundation for my independent research program in addiction science.

**Candidate Background**

**Predoctoral training.** My interest in the treatment of youth behavioral health problems is longstanding. I majored in Psychology and Middle East Studies as an undergraduate at Northwestern University, which shaped my commitment to a career in youth behavioral health research. To further develop my interests in social determinants of health, I pursued a Master’s in Public Health at The University of Illinois at Chicago with concentrations in epidemiology and global health. During my public health training, I gained direct insight into the health inequities youth from underserved neighborhoods face through field experiences in an urban health service system. Motivated to continue addressing behavioral health inequities in youth, I completed a PhD in Child Clinical Psychology from the University of Miami, where I worked with Amanda Jensen-Doss, PhD, an expert in children’s mental health services research. My clinical training included being an evaluator on a randomized control effectiveness trial for youth with anxiety and depression receiving treatment in community mental health clinics (R01MH106536, R01MH106657). This training provided me with foundational skills in the methods used to analyze how and for whom behavioral interventions are most effective. This work also made me feel both enthusiastic about the promise of behavioral interventions to reduce inequities and driven to better understand *why* interventions do not produce equitable, significant change for all youth. As a result, I developed a keen interest in exploring the impact of neighborhood characteristics, often overlooked in clinical trials, on behavioral health intervention outcomes. With funding support from the University of Miami Dean’s Dissertation Fellowship, I combined clinical data from my mentor’s clinical effectiveness trial and U.S. Census data to investigate the relationship between zip codes and anxiety and depression symptoms among treatment seeking youth. I found that youth from underserved neighborhoods presented to treatment with disproportionally greater anxiety and depression symptoms than did youth from more advantaged neighborhoods. Consequently, I wanted to move beyond zip codes, to understand how youth and parent perceptions of neighborhood factors may affect response to intervention, with the ultimate goal of improving behavioral interventions to meet the unique needs of underserved youth and families.

**TAIs for substance use and behavioral health.** During my pre-doctoral clinical internship year, I conducted brief substance use assessments for youth admitted to the UCLA Resnick Neuropsychiatric Hospital inpatient and partial hospitalization programs via telehealth services. I witnessed firsthand the potential of technology to enhance the reach of effective behavioral health interventions for underserved youth and families who often face heighted barriers to treatment access (e.g., high cost, limited proximity to service providers). This year-long, immersive clinical experience working with adolescents in intensive behavioral health services launched me towards a new area of research interest—leveraging technology to improve youth substance use outcomes at scale. Eager to pursue research training in this area, I accepted a postdoctoral research fellowship at Northwestern University Feinberg School of Medicine’s Center for Dissemination and Implementation Science under the mentorship of Dr. Sara Becker, an expert in addiction and implementation science, and my Primary Mentor on this Diversity Supplement proposal. My postdoctoral research aims to improve the effectiveness of youth substance use interventions, with an emphasis on advancing health equity for underserved youth and families. In nine months, my postdoctoral work has led to two first authored publications currently under review and two presentations at the Association for Behavioral and Cognitive Therapies National Conference. This year, I also received the Journal of Clinical Child and Adolescent Psychology Future Directions Launch Award and received early indication of intent to fund my NIH Loan Repayment Program Award: **both awards I received recognize promising early career researchers with exceptional potential for an independent research career**. My postdoctoral fellowship has ignited my enthusiasm for using TAIs to improve youth substance use outcomes and if awarded, this Diversity Supplement will facilitate invaluable training in key methodological and content areas to support my continued career development towards being a fully independent investigator.

**Career Goals and Objectives:** My interdisciplinary training in public health and clinical science has instilled all the requisite capabilities, drive, and skills to launch an independent program of research. To date, I have contributed to 18 publications (11 first author), 1 book chapter, and 9 conference symposia/invited presentations. I have 3 manuscripts under review (2 first author), and several co-author manuscripts in preparation with Drs. Becker, Helseth, Rosales, and Janssen, who are all co-investigators on the parent grant.

**To achieve my long-term goal of becoming an independent investigator I require specialized training in three key areas:** 1) Conducting pragmatic clinical effectiveness trials of TAIs with youth substance use populations; 2) Evaluating the influence of TAIs on equitable access to and engagement in substance use care; and 3) Using multi-level models for longitudinal data to analyze response to TAIs. Achieving these career development goals will help me launch an independent research program focused on improving substance use interventions for underserved youth and families. **Table 2** depicts a plan for transition to independence by clarifying my existing expertise and career development plan as they map on to my long-term career goals.

**Specific Career Development Activities**

Central to all three of the specific career development aims in this Diversity Supplement is need for hands-on mentorship specific to addiction science. As part of my involvement in the parent grant and under Dr. Becker’s mentorship, I will shadow investigators delivering gold-standard clinical assessments for youth substance use, such as the Global Appraisal of Individual Needs^34^ and will observe delivery of the Parent SMART intervention. Exposure to evidence-based substance use assessments and interventions will greatly strengthen my understanding of how to conduct clinical research with high-risk youth and their families. Additionally, participating in weekly team meetings with the parent grant team and conducting site visits to the residential treatment facility with Dr. Becker will foster the research skills required to conduct clinical interventions with youth who use substances and their families.

**Table 2: Plan for Transition to Independence for the Candidate**

Career

Independent Research Program

| **Existing Expertise** | **Career Development Plan** | **Long-Term Career Goals** |
| --- | --- | --- |
| Extensive experience evaluating the effectiveness of behavioral treatment for youth diagnosed with anxiety and depression receiving services in community mental health clinics | Hands-on exposure to participant recruitment methods, data collection and management strategies, and staff supervision skills required to conduct clinical effectiveness trials of TAIs with youth who use substances | **(Research Training Goal 1; Primary Mentor: Sara Becker, PhD)**  Build expertise in conducting pragmatic effectiveness clinical trials of TAIs with youth that use substances |
| Conducted brief substance use assessments via telehealth in behavioral health service settings and learned first-hand about the potential of technology to expand the reach of behavioral health services | In-depth training in the theories used to evaluate equitable access to and in engagement in TAIs (e.g., leading digital health equity models), identifying indicators of successful outcomes in diverse populations, and ethical/legal issues (e.g., privacy, data security) | **(Research Training Goal 2;**  **Secondary Mentors: Sarah Helseth, PhD and Robert Rosales, PhD, LCSW)**  Gain knowledge in role of TAIs in increasing equitable access to and engagement in substance use care |
| A foundation in linear regression models and muti-dimensional measurement of neighborhood characteristics using U.S. Census indicators | Practice integrating multi-level and multi-reporter data in longitudinal models to assess response to substance use TAIs (e.g., a latent growth curve to test multi-level mediation) | **(Research Training Goal 3; Secondary Mentor: Tim Janssen, PhD)**  Become a leader in the application of quantitative skills in multi-level data analysis to assess response to substance use TAIs |

These specific career development experiences in addiction science will be supplemented by the stellar research infrastructure and training opportunities available at the Northwestern University Feinberg School of Medicine. For instance, I will attend the weekly seminar series in the implementation of addiction treatment co-sponsored by the Northwestern’s Prevention Services Methodology Group and the Center for Dissemination and Implementation Science at Stanford University (a P50 grant on which my Primary Mentor is core faculty). Example talks given this past year that are directly relevant to the current proposal include: A Proposed Framework for Design Trials Evaluating the Effectiveness of Digital Interventions for Substance Use; Alliances to Disseminate Addiction Prevention and Treatment; and Development of an Integrated Implementation Determinant Framework for Shared Learning to Promote Health Equity. I will also work with my mentorship team to select relevant seminars from the Center for Behavioral Information Technology (CBITs), which offers seminars on TAIs and other technological innovations monthly, and from the Institute for Public Health and Medicine Annual Series, which offers seminars on a range of public health topics weekly. Additionally, I will participate in Grant Writing Coaching Groups offered by Northwestern’s Faculty Affairs Office, in preparation for a successful NIDA R34 application to adapt the Parent SMART intervention to meet the needs of youth and families living in underserved neighborhoods. These novel grant writing groups for early career faculty are led by Rick McGee, PhD, and meet for 90 minutes every week for four months to iteratively develop an NIH Research proposal with group feedback. In addition to seminars and grant writing groups, I will also utilize the Northwestern University Clinical and Translational Science Institute’s grant studio consultations, which support interdisciplinary federal grant submissions, and the Biostatistics Collaboration Center, which provides methodological support for research proposals. Together, the expertise of my mentorship team and the career development resources available at Northwestern University are a strong foundation for training in addiction science and to launch an independent program of research in improving the effectiveness of TAIs.

If awarded, this Diversity Supplement will also provide me with protected time to prepare peer reviewed manuscripts and the necessary funds to attend national conferences and network with leaders in the addiction field. My Primary Mentor and I have outlined a detailed plan for manuscript submissions based on the findings of the Diversity Supplement and our existing collaborations. Specifically, I will lead two first-authored manuscripts over the two-year funding period. The first manuscript will focus on the theoretical importance of assessing neighborhood perceptions in substance use research. The second manuscript will describe the empirical findings from the Diversity Supplement. I will also contribute to four co-authored manuscripts in collaboration with Dr. Sara Becker and the parent grant team (2 co-author manuscripts will be submitted yearly). Example co-authors manuscript topics include: the main outcome results from the parent grant, the effects of Parent SMART on co-occurring mental health problems in youth, and qualitatively characterizing parent feedback on the Parent SMART intervention. Additionally, I will attend two national conferences per year (e.g., Association for Behavioral and Cognitive Therapies, Collaborative Perspectives on Addiction, The Society for Implementation Research Collaboration), to present my work and develop collaborations with leaders in the addiction science field to prepare future grant submissions. Manuscript writing, attendance at national conference, and grantsmanship are vital professional development activities to support my program of research and transition from postdoctoral fellow to an independent investigator.

**Timeline for Research and Career Development Activities**

| **Table 3. Research and Career Development Activities by Year** | **Year 1**  Beginning June 2023 | | | | | | | | | | | | **Year 2**  Beginning June 2024 | | | | | | | | | | | |
| --- | --- | --- | --- | --- | --- | --- | --- | --- | --- | --- | --- | --- | --- | --- | --- | --- | --- | --- | --- | --- | --- | --- | --- | --- |
|  | 1 | 2 | 3 | 4 | 5 | 6 | 7 | 8 | 9 | 10 | 11 | 12 | 1 | 2 | 3 | 4 | 5 | 6 | 7 | 8 | 9 | 10 | 11 | 12 |
| **Research Activities** | | | | | | | | | | | | | | | | | | | | | | | | |
| Diversity Supplement Data Collection: Update parent grant assessment plan with neighborhood characteristics, submit IRB amendment, train research staff and collect data from parents and youth |  |  |  |  |  |  |  |  |  |  |  |  |  |  |  |  |  |  |  |  |  |  |  |  |
| Diversity Supplement Data Analysis: Explore variance in neighborhood characteristics, identify salient predictors for analysis, generate analytic code for aims, explore multi-level mediation |  |  |  |  |  |  |  |  |  |  |  |  |  |  |  |  |  |  |  |  |  |  |  |  |
| Conduct mentored site visits to residential youth substance treatment facility with Dr. Becker (2x/year) |  |  |  |  |  |  |  |  |  |  |  |  |  |  |  |  |  |  |  |  |  |  |  |  |
| Participate in weekly team research meetings on progress of the parent grant |  |  |  |  |  |  |  |  |  |  |  |  |  |  |  |  |  |  |  |  |  |  |  |  |
| Shadow investigators conducting the family assessments and delivering Parent SMART |  |  |  |  |  |  |  |  |  |  |  |  |  |  |  |  |  |  |  |  |  |  |  |  |
| **Career Development Activities** | | | | | | | | | | | | | | | | | | | | | | | | |
| **Mentorship:** Weekly individual meetings with Primary Mentor Dr. Becker to review progress on Diversity Supplement research aims and career development milestones |  |  |  |  |  |  |  |  |  |  |  |  |  |  |  |  |  |  |  |  |  |  |  |  |
| **Mentorship:** Monthly meetings with co-mentors to develop data analysis plan, conduct analyses, and interpret findings |  |  |  |  |  |  |  |  |  |  |  |  |  |  |  |  |  |  |  |  |  |  |  |  |
| **Manuscript Writing:** Prepare *two* first-authored manuscripts based on the Diversity Supplement |  |  |  |  |  |  |  |  |  |  |  |  |  |  |  |  |  |  |  |  |  |  |  |  |
| **Manuscript Writing:** Contribute to *four* co-authored manuscripts, in collaboration with mentorship team (2 per year) |  |  |  |  |  |  |  |  |  |  |  |  |  |  |  |  |  |  |  |  |  |  |  |  |
| **Grant Writing:** Participate in grant writing coaching groups for early career faculty at Northwestern University |  |  |  |  |  |  |  |  |  |  |  |  |  |  |  |  |  |  |  |  |  |  |  |  |
| **Grant Writing:** Prepare a R34 proposal on adapting Parent SMART to meet the needs of families in underserved neighborhoods |  |  |  |  |  |  |  |  |  |  |  |  |  |  |  |  |  |  |  |  |  |  |  |  |
| **Seminars:** Attend monthly seminars offered through Northwestern’s Prevention Services Methodology Group and Center for Behavioral Information Technology |  |  |  |  |  |  |  |  |  |  |  |  |  |  |  |  |  |  |  |  |  |  |  |  |
| **Conference Presentations:** Attend two national conferences per year |  |  |  |  |  |  |  |  |  |  |  |  |  |  |  |  |  |  |  |  |  |  |  |  |
| **Networking:** Develop local and national collaborations with leaders in the substance use field for future grant proposals |  |  |  |  |  |  |  |  |  |  |  |  |  |  |  |  |  |  |  |  |  |  |  |  |
| **In-Person Coursework:** “Northwestern University’s Taking Responsibility for the Responsible Conduct of Research” course |  |  |  |  |  |  |  |  |  |  |  |  |  |  |  |  |  |  |  |  |  |  |  |  |

The funding support period for the Diversity Supplement is 2 years. **Table 3** above contains a timeline of research and career development activities that are foundational to launching an independent research program during the supplement period. Shaded boxes denote when the activity will occur.

**MENTORING PLAN**

My interdisciplinary expertise in public health and clinical psychology is a solid skillset on which to build a research program focused on improving substance use interventions for youth and families. Combined with the strengths of my mentoring team and the resources available at Northwestern University’s Feinberg School of Medicine, I am well positioned to study the role of neighborhood characteristics on the effectiveness of TAIs for substance use. This work will enable the field to move beyond individual- and family-level sociodemographic factors and will promote the consideration of multi-level health equity determinants in substance use intervention research. To meet my research aims and career development goals, I have selected an outstanding interdisciplinary team of scientists with expertise in conducting clinical effectiveness trials of TAIs with youth substance use populations, evaluating the equitably delivery of substance use TAIs, and multi-level longitudinal data analysis. Of note, Drs. Becker, Helseth, Rosales, and Janssen are all Co-Investigators on the parent grant and have longstanding working relationships as demonstrated by their grant and collaborative publication records. I intentionally crafted a mentoring plan that catered to the strengths of the existing investigative team to ensure that I have the scaffolding needed for ongoing support and mentorship: the investigative team meets weekly and I will be fully integrated into those meetings, in addition to the one-on-one meetings outlined below, to ensure frequent touchpoints with the team. Over the two years, I will work closely with my mentorship team to review progress on the Diversity Supplement and my career development goals.

**Mentorship Team**

**Sara Becker, PhD (Primary Mentor: Conducting pragmatic clinical effectiveness trials of TAIs with youth substance use populations):** Dr. Sara Becker is the Principal Investigator of the Parent Grant and my Primary Mentor on this Diversity Supplement. She serves as the Inaugural Director of the Center for Dissemination and Implementation Science at Northwestern University Feinberg School of Medicine. Since 2012, Dr. Becker has been the Principal Investigator of 11 grants from the National Institutes of Health, Substance Abuse and Mental Health Services Administration, President’s Emergency Plan for AIDS Relief, and Agency for Healthcare and Research Quality. As such, she has extensive experience working with large, multidisciplinary teams, and cultivating relationships with community partners. As my Primary Mentor, Dr. Becker will draw on her extensive experience as a mentor and teacher in the fields of addiction science and implementation science. Dr. Becker is currently primary mentor or co-mentor on 4 active career development awards (3 NIH, 1 Society of Emergency Medicine). She also frequently mentors undergraduates, graduate students, postdoctoral fellows, and residents. Her publication record demonstrates her commitment to publishing with mentees. Over the nine months of my postdoctoral fellowship, Dr. Becker and I have collaborated on several manuscripts and have had weekly face-to-face meetings to discuss my research and career development goals.

Dr. Becker will help prepare my successful transition to an independent addiction scientist by providing me with immersive training in conducting youth substance use research. Across the two years of the Diversity Supplement, Dr. Becker and I will continue to meet weekly for individual mentorship meetings to discuss progress towards Diversity Supplement research aims, monitor my active participation in career development activities, and collaboratively problem solve roadblocks as they arise. During these individual meetings, we will also discuss ethical issues related to conducting research with high-risk youth substance use populations, review drafts of my planned manuscripts and grant submissions, and identify additional opportunities for presentations of my work beyond national conference submissions (e.g., brown bag presentations at the Northwestern Feinberg School of Medicine). To foster my development as an independent investigator, l will be fully integrated into the parent grant, including both research and clinical activities. I will fully participate in weekly research team meetings for the parent project, where Dr. Becker will provide me with hands on guidance regarding the participant recruitment methods, data collection and management strategies, and staff supervision skills required to conduct clinical effectiveness trials with substance using youth. Dr. Becker will also provide me with the opportunities to apply these skills by inviting me to co-lead meetings with our residential treatment program partners, providing hands-on supervision as I immerse myself in shadowing activities for the parent grant, and providing feedback as I develop skills to supervise a research study assistant in data management. As indicated in her biosketch and letter of attestation, Dr. Becker is dedicated to serving as a primary mentor on this Diversity Supplement and has been actively engaged in each step of the supplement writing process, including the selection of mentors and the development of a detailed career development plan.

**Sarah Helseth, PhD (Evaluating the influence of TAIs on equitable access to and engagement in substance use care):** Dr. Sarah Helseth is a health services researcher and licensed clinical psychologist. She is currently an Assistant Professor of Psychiatry and Behavioral Science at Northwestern University and is core faculty at the Center for Dissemination and Implementation Science at Northwestern University Feinberg School of Medicine. Dr. Helseth’s research aims to reduce the impact of behavioral health problems through the development and dissemination and effective technology-assisted interventions, particularly among underserved populations. She is currently the PI of a 5-year, NIDA-funded Early Career Award (K23DA048062) to develop and implement digital health technologies to reduce marijuana use among court-involved, non-incarcerated youth. She is also a Co-Investigator on the parent project for which this Diversity Supplement is being submitted.

Dr. Helseth will provide critical mentorship in the theories and methods used to evaluate the effectiveness of TAIs, the ethical and legal issues involved in conducing digital health service research (e.g., privacy, data security), and support me in identifying indicators of successful engagement with Parent SMART (i.e., use of Parenting Wisely, attendance at telehealth coaching sessions, use of the parenting network forum). Dr. Helseth will also provide clinical supervision and oversight as I train to deliver the Parent SMART intervention to families enrolled in the project. These skills are necessary to accomplish the research aims of the Diversity Supplement and will support my long-term career goal of developing a research program focused on improving substance use outcomes for underserved youth. Dr. Helseth is committed to serving as an on-site secondary mentor on the Diversity Supplement and will provide me with individual mentorship through monthly face-to-face research meetings during Years 1 and 2 of the Diversity Supplement.

**Robert Rosales, PhD, LCSW (Evaluating the influence of TAIs on equitable access to and engagement in substance use care):** Dr. Robert Rosales, PhD, LCSW, is an Assistant Professor in the Center for Alcohol and Addiction Studies at the Brown University School of Public Health. Dr. Rosales studies the effects of minority stress (e.g., discrimination) and sociocultural protective factors (e.g., community support) on behavioral health issues (e.g., alcohol use). He also studies the multilevel predictors of quality health care access. For instance, he has published research on how the Affordable Care Act is associated with racial and ethnic minorities’ access to behavioral health and integrated care. He is also currently the PI on a K08 Award from the National Institute of Minority Health and Health Disparities (NIMHD) titled “Socio-Cultural Protective and Risk Factors of Alcohol Use among Non-Hispanic White and Hispanic Sexual Minority Youth (K08 MD015289).” Dr. Rosales’ is an expert in substance use in youth populations, longitudinal data analysis, and health equity measurement. He is also a Co-Investigator on the parent project for which this Diversity Supplement is being submitted.

Given his expertise as a health equity scholar, Dr. Rosales will provide me with guidance in evaluating how TAIs can be used to promote health equity in substance use care. Specific considerations include inclusion and representation of diverse youth in study samples of TAI effectiveness trials, tailoring of TAIs to meet the unique needs of youth and families living in underserved neighborhoods, and strategies to support individuals who may have less familiarity with technology to facilitate TAI engagement. As I move forward with data collection and analysis, Dr. Rosales has agreed to meet with me monthly via teleconference to help select health equity training seminars at Northwestern University relevant for the Diversity Supplement and to provide me with opportunities to apply lessons learned (e.g., discuss the role of neighborhoods in maintaining health inequities in TAI response). Dr. Rosales is a remote mentor, but we will interact weekly in an hour-long weekly team meeting with Drs. Becker, Helseth, Rosales, and research staff about the R37 parent project, in addition to our monthly mentorship meetings.

**Tim Janssen, PhD (Quantitative Methods: Longitudinal multi-Level modeling to assess response to substance use intervention):** Dr. Tim Janssen is an Assistant Professor in the Center for Alcohol and Addiction Studies at the Brown University School of Public Health. He is Principal Investigator of two NIH grants that investigate the development of alcohol use initiation and escalation in adolescents and young adults (K01AA026335 and R21AA025716). Dr. Janssen’s research interests include advancing the state of analytical techniques to improve the accuracy and reliability of empirical evidence. Notably, his work capitalizes on structural and multi-level modeling to accurately represent developmental changes in mechanisms predicting substance use initiation and escalation. He is also a Co-Investigator on the parent project for which this Diversity Supplement is being submitted.

Dr. Janssen will provide key oversight in the quantitative methods required to integrate and analyze multi-level self-report data (e.g., fit of a latent growth curve to test multi-level mediation versus a piece-wise model). Although I have foundational knowledge in linear regression models, testing for multi-level mediation is a quantitative skill beyond the scope of my existing expertise, and necessary to assess response to TAIs. Dr. Janssen will provide hands-on mentorship as I integrate multi-level data collection of community-level variables with individual- and family-level sociodemographic factors. He will meet with me monthly during Years 1 and 2 of the Diversity Supplement via teleconference and provide me with mentorship in effective data cleaning strategies, directed readings on longitudinal data analysis, and tools for handling missing data in multi-level analysis. Dr. Janssen is committed to reviewing analysis code I independently generate to facilitate follow-through of the analytic plan and will also support me in interpreting results from multi-level longitudinal models. Although Dr. Janssen is a remote mentor, we will have weekly opportunities to interact given we will both participate in an hour-long weekly team meeting with Drs. Becker, Helseth, Rosales, and research staff about the R37 parent project.

**Guidance in the Ethical Conduct of Research**

I have completed online coursework through the Collaborative Institutional Training Initiative (CITI) at Northwestern University on the responsible conduct of research (RCR) within human subject’s research in social and behavioral research contexts. I will continue training in RCR as a core component of this Diversity Supplement. This includes in-person participation in didactic coursework from Northwestern University Feinberg School of Medicine’s Taking Responsibility for the Responsible Conduct of Research Course (NUCATS RCR), ongoing CITI training, and individualized mentorship in research ethics from my Primary Mentor and secondary mentors. Northwestern’s RCR training was specifically designed to address the National Institutes of Health guidelines for training in RCR. Each RCR seminar is taught by faculty from the Northwestern University Feinberg School of Medicine and representative topics include research misconduct, conflicts of interest, publication practices, mentoring, data management, and ethics in human subjects research. My progress in RCR will be documented on a consistent basis through regular course attendance, assignment completion, and via annual self-evaluation forms that will be shared with my mentorship team.

The expertise of my interdisciplinary mentorship team strategically aligns with my short-term training aims and long-term career development goals. Individual mentorship meetings and team research meetings, supplemented with the resources available at Northwestern University Feinberg School of Medicine (see Career Development Plan), will enable me to acquire the practical knowledge, conceptual competencies, and methodological skills required to develop an independent research program focused on improving the effectiveness of substance use interventions for underserved youth and families. **Through the critical support provided by the Diversity Supplement during a pivotal point in my career, I will attain the proficiencies necessary to pursue a career as an independent investigator, which will consequently increase the number of underrepresented scientists conducting youth substance use research.**
